# Small‐field dosimetry of TrueBeam^TM^ flattened and flattening filter‐free beams: A multi‐institutional analysis

**DOI:** 10.1002/acm2.12791

**Published:** 2019-12-09

**Authors:** Yuichi Akino, Hirokazu Mizuno, Masaru Isono, Yoshihiro Tanaka, Norihisa Masai, Toshijiro Yamamoto

**Affiliations:** ^1^ Oncology Center Osaka University Hospital Suita Osaka Japan; ^2^ Department of Medical Physics and Engineering Osaka University Graduate School of Medicine Suita Osaka Japan; ^3^ Department of Radiation Oncology Osaka International Cancer Institute Osaka Japan; ^4^ Department of Radiation Therapy Japanese Red Cross Society Kyoto Daiichi Hospital Kyoto Prefecture Japan; ^5^ Miyakojima IGRT Clinic Miyakojima‐ku Osaka Japan; ^6^ Department of Radiology Saiseikai‐Noe Hospital Osaka Japan

**Keywords:** beam data commissioning, flattening filter‐free beams, small‐field dosimetry, TrueBeam

## Abstract

**Purpose:**

Detector‐dependent interinstitutional variations of the beam data may lead to uncertainties of the delivered dose to patients. Here we evaluated the inter‐unit variability of the flattened and flattening filter‐free (FFF) beam data of multiple TrueBeam (Varian Medical Systems) linear accelerators focusing on the small‐field dosimetry.

**Methods:**

The beam data of 6‐ and 10‐MV photon beams with and without flattening filter measured for modeling of an iPLAN treatment planning system (BrainLAB) were collected from 12 institutions — ten HD120 Multileaf Collimator (MLC) and two Millennium120 MLC. Percent‐depth dose (PDD), off‐center ratio (OCR), and detector output factors (*OF*_det_) measured with different detectors were evaluated. To investigate the detector‐associated effects, we evaluated the inter‐unit variations of the *OF*_det_ before and after having applied the output correction factors provided by the International Atomic Energy Agency (IAEA) Technical Reports Series no. 483.

**Results:**

PDD measured with a field size of 5 × 5 mm^2^ showed that the data measured using an ionization chamber had variations exceeding 1% from the median values. The maximum difference from median value was 2.87% for 10 MV photon beam. The maximum variations of the penumbra width for OCR with 10 × 10 mm^2^ field size were 0.97 mm. The *OF*_det_ showed large variations exceeding 15% for a field size of 5 × 5 mm^2^. When the output correction factors were applied to the *OF*_det_, the variations were greatly reduced. The relative difference of almost all field output factors were within ± 5% from the median field output factors.

**Conclusion:**

In this study, the inter‐unit variability of small‐field dosimetry was evaluated for TrueBeam linear accelerators. The variations were large at a field size of 5 × 5 mm^2^, and most occurred in a detector‐dependent manner.

## 1 INTRODUCTION

Stereotactic radiotherapy (SRT) has shown excellent clinical outcomes for the treatment of metastatic brain tumors^1^, ^2^, ^3^ and also for extracranial tumors, such as those of lungs, liver, and prostate.^4^, ^5^ Although photon beams with small field sizes are often used for such treatments, accurate small‐field dosimetry remains challenging.^6^, ^7^, ^8^ Beam‐related causes of variations in beam characteristics include lateral charged‐particle disequilibrium, partial occlusion of the direct beam source,^9^ and change to the energy spectrum of photons,^10^ whereas detector‐related causes include volume‐averaging effects as well as detector and shielding materials affecting the perturbation of the charged‐particle fluence and the mass electronic stopping power.^11^, ^12^ Many detectors for small‐field dosimetry, such as shielded and unshielded diodes, diamond detectors, and plastic scintillators, have different characteristics, such as sensitive volumes, shielding materials, and detector materials affecting the perturbations and stopping power ratio.^13^, ^14^, ^15^

A treatment planning system (TPS) uses scanning and non‐scanning measured data for modeling of the x‐ray beam data. However, many do not require data of very small field sizes of ≤10 mm for beam modeling. For example, field sizes < 30 × 30 mm^2^ do not have a significant impact on the beam modeling of Eclipse (Varian Medical Systems, Palo Alto, CA) TPS,^16^ and tuning of parameters is needed for accurate calculation of small fields.^17^ Therefore, most institutions do not acquire such small‐field data or measure small fields with each institutional protocol, which results in difficulties in the evaluation of the inter‐variability among institutions. The iPLAN (BrainLAB, Munich, Germany) TPS requires very small‐field data for beam modeling. Therefore, institutions equipped with the iPLAN TPS acquire small‐field data with the same protocol. Akino et al.^18^ previously evaluated 19 beam datasets of a Novalis Tx (Varian Medical Systems and BrainLAB) linear accelerator (linac) measured for modeling iPLAN TPS and reported large variations especially for a field size of 5 × 5 mm^2^. If such variations were not machine‐specific, but rather caused by the selection of detectors or operator‐associated uncertainties, such variations will result in discrepancies in the delivered dose to patients.

Alfonso et al.^19^ proposed a new formula for small‐field dosimetry, as updated in the International Atomic Energy Agency (IAEA) Technical Reports Series no. 483 (TRS‐483).^8^ In this formalism, the output correction factor ($k_{Q_{\text{clin}},Q_{\text{msr}}}^{f_{\text{clin}},f_{\text{msr}}}$) is applied to the ratio of detector readings (detector output factor: *OF*_det_) to obtain the corrected field output factor ($\Omega_{Q_{\text{clin}},Q_{\text{msr}}}^{f_{\text{clin}},f_{\text{msr}}}$). Figure S1 shows an example of *OF*_det_ corrected by the $k_{Q_{\text{clin}},Q_{\text{msr}}}^{f_{\text{clin}},f_{\text{msr}}}$ factors. Akino et al.^18^ reported that the interinstitutional variations of the *OF*_det_ measured with small field sizes of ≤10 mm were reduced by applying the factors $k_{Q_{\text{clin}},Q_{\text{msr}}}^{f_{\text{clin}},f_{\text{msr}}}$, indicating that the variations were primarily associated with the detector selection.

The TrueBeam (Varian Medical Systems) is one of the latest generations of linacs. This machine and recent technologies have enabled reduction of the treatment time by use of flattening filter‐free (FFF) beams,^20^ volumetric‐modulated arc therapy, and linac‐based single‐isocenter noncoplanar techniques.^21^, ^22^ Although a few studies have investigated the inter‐unit variability of the TrueBeam,^23^, ^24^, ^25^ none has yet explored very small‐field (≤10 mm) dosimetry of the TrueBeam. Here, we evaluated the flattened and FFF beams of the TrueBeam machines of multiple institutions.

## 2 METHODS AND MATERIALS

### 2.1 Beam data collection

The beam data of 12 linacs — ten TrueBeam STx machines with a HD120 Multileaf Collimator (MLC) and two TrueBeam machines with Millennium120 MLC — were collected from 11 institutions. Under institutional agreement, the beam data for modeling iPLAN TPS were provided by the TPS vendor. The data were collected between August and November in 2017. All institutions submitted their beam data to the vendor before IAEA TRS‐483 was published, indicating that the submitted data were measured data without output corrections. In this study, two types of data were collected: (i) Microsoft Excel spreadsheets for modeling of pencil beam algorithms and (ii) binary files submitted to the TPS vendor for modeling of the Monte Carlo algorithm. For modeling of pencil beam algorithms, the measured data are usually input into vendor‐provided spreadsheets and copied into the TPS. The spreadsheets contain various data, including collimator transmission, percentage depth dose (PDD), scatter factor calculated as the ratio of detector readings (*OF*_det_), diagonal profile, transversal profile, and dynamic leaf shift which was the MLC parameter and was calculated from measurements of moving slit beams with gap sizes from 1 mm to 100 mm. The detectors used for measurements were also noted in the spreadsheets. The field sizes were defined by the machine setting of MLC opening for all field sizes. For 5 × 5 mm^2^ MLC field size, the jaws field size was 8 × 8 mm^2^. For 10–40 mm field sizes, the jaws field sizes were 2 mm larger than the MLC field sizes for both X and Y axes. For field sizes >40 mm, the jaws were located at the same position to the MLC. Details of the data collected for pencil beam algorithm are described elsewhere.^18^ The detectors used for measurements were also recorded in the Excel files. Although all institutions noted two or more detectors used for measurements in the Excel files, some institutions did not noted the field sizes for each detector. The following detectors were used for the smallest field size: EDGE (Sun Nuclear Corp., Melbourne, FL); Diode E, Model 60017 Dosimetry Diode Type E (PTW‐Freiburg GmbH, Freiburg, Germany); Diode SRS, Model 60018 Dosimetry Diode SRS (PTW); microDiamond, Model 60019 Synthetic Diamond Detector (PTW); PinPoint ionization chamber, Model 31016 (PTW); SFD (IBA Dosimetry GmbH, Schwarzenbruck, Germany); and CC01 ionization chamber (IBA). In total, 11 and 12 Excel datasets of flattened and FFF beams were collected, respectively. Because the measured data of the off‐center ratio (OCR) are needed only for the Monte Carlo algorithm, the spreadsheets did not contain OCR. Therefore, the binary data for Monte Carlo beam modeling were imported into an iPLAN TPS to extract the OCR. For some PDD and OCR whose resolutions were different from the default of the spreadsheets, the resolutions were corrected using resampling with linear interpolation to calculate the median value and variation at each data point. The PDD and *OF*_det_ were collected at a 100 cm source‐to‐surface distance (SSD), whereas the OCR were collected at 90 cm SSD. The *OF*_det_ and OCR were measured at 10 cm depth. Some institutions did not have the option of a Monte Carlo algorithm, which resulted in a limited collection of OCR. Especially for FFF beams, only the recent version of the iPLAN TPS (version 4.5.4 or later) supports the Monte Carlo calculation. In total, seven and three OCR sets of flattened and FFF beams were collected, respectively. At one institution, the OCR of FFF beams were collected later using a microDiamond detector. Table 1 shows the detector types used for measuring PDD, *OF*_det_, and OCR, with the number of institutions.

**Table 1:** Types of detectors and number of institutions used for the measurements.

Detector	Type	Sensitive volume	Flattened			FFF		
			PDD	*OF*_det_	OCR	PDD	*OF*_det_	OCR
EDGE	Shielded diode	0.8 × 0.8 mm^2^	5	7	4	5	7	1
PTW 60017	Unshielded diode	1.13 mm φ	2	2	2	2	2	0
PTW 60018	Unshielded diode	1.13 mm φ	0	0	0	0	1	0
SFD	Unshielded diode	0.6 mm φ	1	0	0	1	0	0
PTW 31016	Ionization chamber	0.016 cm^3^	1	0	0	1	0	0
CC01	Ionization chamber	0.01 cm^3^	1*	1	1	1*	1	0
PTW 60019	Diamond	2.2 mm φ	1	1	0	2	1	2
Total			11	11	7	12	12	3

Abbreviations: FFF, flattening filter‐free beams; OCR, off‐center ratio; *OF*_det_, ratio of detector readings; PDD, percentage depth dose.

* This institution used the IBA CC04 ionization chamber for PDD measurements of a field size ≥ 10 mm^2^.

### 2.2 Analysis of the scanning data

For PDD, the data measured with field sizes of 5 × 5 and 10 × 10 mm^2^ were evaluated. For two linacs with the Millennium120 MLC, the data for a field size of 5 × 5 mm^2^ were not collected. All PDD were normalized at the peak depth (*d*_max_). To evaluate interinstitutional variability, a median value of all detector data was calculated for each data point, and the difference from the median value was calculated. For OCR, the data measured with field sizes of 10 × 10 mm^2^ were evaluated. All data were normalized at the central axis, and the full width at half maximum (FWHM) value and the penumbrae width, as defined by positions of the 20–80% profile, were calculated. The offset of the beam center was calculated as the distance between the center of the FWHM and the central axis, and the values were within ±0.23 mm. Although the corrections did not affect the calculations of the FWHM and penumbra width, the offsets of the OCR were corrected to show the variability. Because the FFF beam profiles are cone shaped, FWMH will not be an appropriate parameter. Fogliata et al.^26^ suggested renormalization. For small field sizes, however, the flattened and FFF beam profiles show very similar shape. Because we evaluated only 10 × 10 mm^2^ field size in this study, we evaluated FWHM with normalization of the profiles at the center. The scanning data of all institutions were resampled with the resolution of 0.5 mm to calculate the median and variability, but any other processing such as smoothing was not used, although the data submitted to the vendor may possibly be processed.

### 2.3 Analysis of the output factors

For *OF*_det_, only the square field sizes were evaluated to simplify the analysis. All data were normalized to a field size of 100 × 100 mm^2^. The minimum field sizes for the HD120 MLC and Millennium120 MLC were 5 × 5 and 10 × 10 mm^2^, respectively. To evaluate the impacts of the detectors on the scatter factors, the *OF*_det_ values were corrected by output correction factors, as summarized in the recent IAEA TRS‐483 report.^8^ For the CC01 and EDGE detectors, the correction factors for 5 × 5 mm^2^ field size were calculated with extrapolation. Although the literature provided the values for the EDGE detector only for field sizes ≥8 × 8 mm^2^, the extrapolated values for 5 × 5 mm^2^ field size were close to those reported previously.^27^, ^28^ Usually, detectors for small‐field dosimetry are not used for measurements of non‐small field sizes because of the polarity effects and energy dependence of the detectors.^29^ These field sizes are often measured with ionization chambers with sensitive volumes of approximately 0.1 cm^3^, and values for small field sizes measured with other detectors are combined with renormalization at an intermediate field size (such as 20–40 mm). In this study, the $k_{Q_{\text{clin}},Q_{\text{msr}}}^{f_{\text{clin}},f_{\text{msr}}}$ factors were divided by the mean values of 20 × 20 mm^2^ and 40 × 40 mm^2^ field sizes to normalize the intermediate field size. For both *OF*_det_ and $\Omega_{Q_{\text{clin}},Q_{\text{msr}}}^{f_{\text{clin}},f_{\text{msr}}}$, the inter‐unit variability was evaluated based on the relative difference, which was defined as the difference between each data point and the median $\Omega_{Q_{\text{clin}},Q_{\text{msr}}}^{f_{\text{clin}},f_{\text{msr}}}$ value divided by the median $\Omega_{Q_{\text{clin}},Q_{\text{msr}}}^{f_{\text{clin}},f_{\text{msr}}}$ value to show the effects of the output corrections. For each beam energy, differences between the *OF*_det_ and $\Omega_{Q_{\text{clin}},Q_{\text{msr}}}^{f_{\text{clin}},f_{\text{msr}}}$ values were compared using Wilcoxon signed rank test using JMP software (ver. 14.0, SAS Institute, Cary, NC), and statistical significance was set at a *P* value of <0.05.

## 3 RESULTS

**Figure 1:**
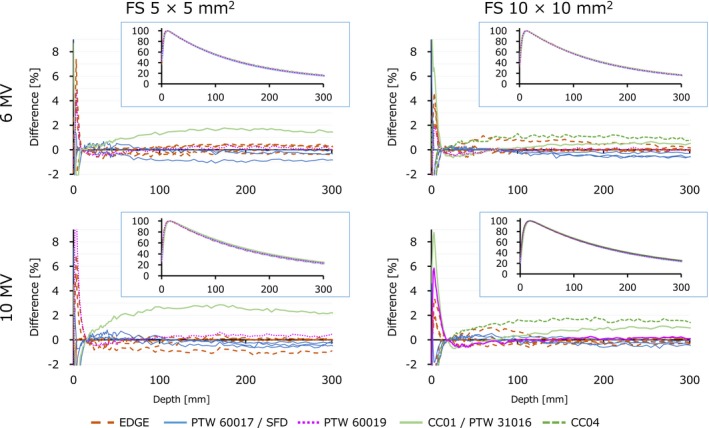
Percentage depth dose (insets) and the difference of each data point from the median curve for flattened two beams and two field sizes. FS, field size.

**Figure 2:**
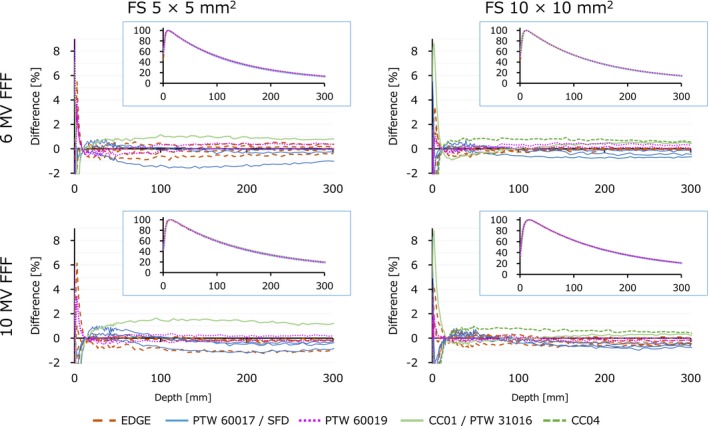
Percentage depth dose (insets) and the difference of each data point from the median curve for FFF two beams and two field sizes. FFF, flattening filter‐free beams; FS, field size.

Figure 1 shows the PDD of the flattened beams. The values subtracted by the mean of each data point were plotted, and the original PDD curves are shown in the insets. At a field size of 5 × 5 mm^2^, one institution using the CC01 ionization chamber had variations exceeding 1%. The maximum difference from median value was 2.87% for 10 MV photon beam. A few data measured with EDGE and Diode E also showed variations slightly exceeding 1%. For a field size of 10 × 10 mm^2^, almost all data were within 1%, although one institution using the CC04 ionization chamber exceeded 1%. The FFF data illustrated in Fig. 2 showed similar results.

**Figure 3:**
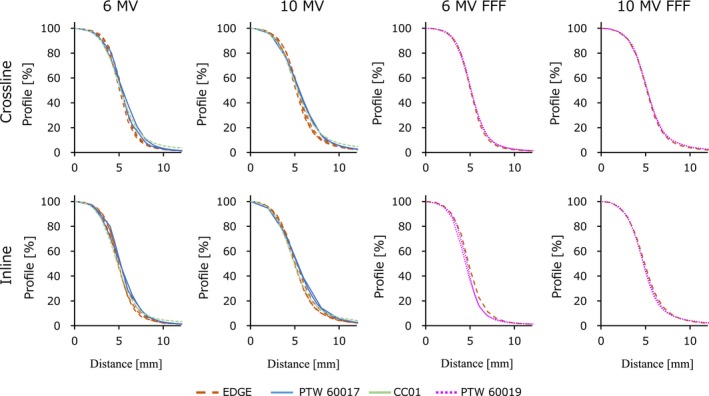
Off‐center ratio profiles for a field size of 10 × 10 mm^2^. FFF, flattening filter‐free beams.

**Figure 4:**
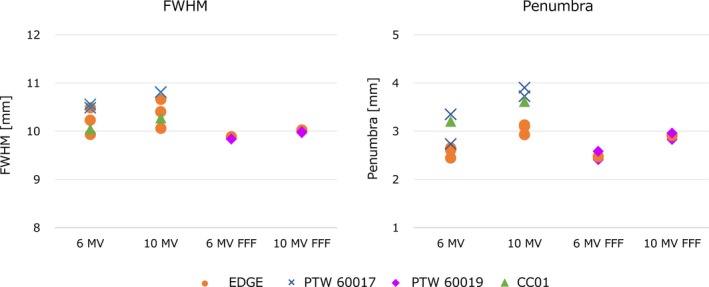
FWHM and penumbra width values of the OCR profiles of a field size of 10 × 10 mm^2^. Both values represent the mean crossline and inline values. For the penumbra values, the mean right and left penumbra widths were evaluated. FFF, flattening filter‐free beams; FWHM, full width at half maximum.

Figure 3 shows the OCR profiles of the flattened and FFF beams measured at a field size of 10 × 10 mm^2^. The center of the profiles was corrected and only the positive side of the axis is shown. Figure 4 shows the FWHM and penumbrae width values. The penumbrae width represents the mean of the left and right values as beam profiles of 20–80%. For both the FWHM and penumbrae width, the mean crossline and inline values were plotted. The maximum variations in FWHM were 0.62 mm, 0.75 mm, 0.05 mm, and 0.06 mm for 6 MV, 10 MV, 6 MV FFF, and 10 MV FFF beams, respectively. Although the data were limited, the penumbrae width measured with the CC01 and diode E seemed slightly wider than those measured with the EDGE and microDiamond. As shown by the FFF data, the EDGE and microDiamond had very similar OCR profiles. The maximum variations in penumbrae were 0.91 mm, 0.97 mm, 0.17 mm, and 0.13 mm for 6 MV, 10 MV, 6 MV FFF, and 10 MV FFF beams, respectively.

**Figure 5:**
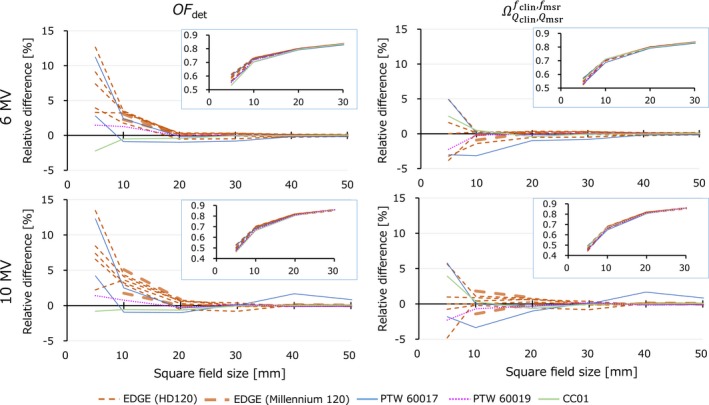
Uncorrected (*OF*_det_, left column) and corrected ($\Omega_{Q_{\text{clin}},Q_{\text{msr}}}^{f_{\text{clin}},f_{\text{msr}}}$, right column) field output factors of the flattened beams (insets) and the relative difference of each value from the median $\Omega_{Q_{\text{clin}},Q_{\text{msr}}}^{f_{\text{clin}},f_{\text{msr}}}$ value.

**Figure 6:**
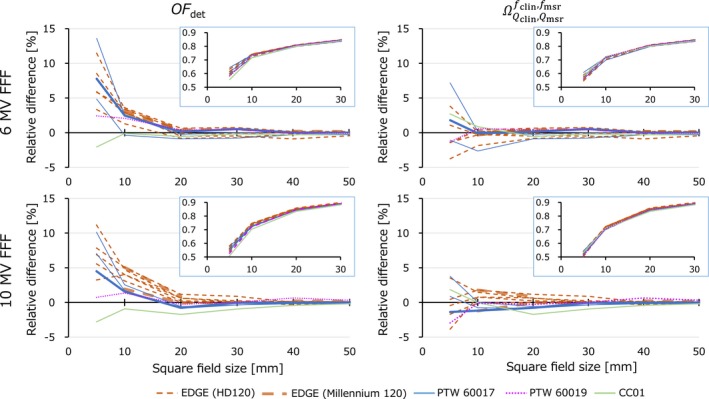
Uncorrected (*OF*_det_, left column) and corrected ($\Omega_{Q_{\text{clin}},Q_{\text{msr}}}^{f_{\text{clin}},f_{\text{msr}}}$, right column) field output factors of the FFF beams (insets) and the relative difference of each value from the median $\Omega_{Q_{\text{clin}},Q_{\text{msr}}}^{f_{\text{clin}},f_{\text{msr}}}$ value. FFF, flattening filter‐free beams.

Figures 5 and 6 show the variations of *OF*_det_ and $\Omega_{Q_{\text{clin}},Q_{\text{msr}}}^{f_{\text{clin}},f_{\text{msr}}}$ relative to the median $\Omega_{Q_{\text{clin}},Q_{\text{msr}}}^{f_{\text{clin}},f_{\text{msr}}}$ value evaluated for flattened and FFF beams, respectively. In the insets, the original *OF*_det_ values provided by each institution were plotted. The $\Omega_{Q_{\text{clin}},Q_{\text{msr}}}^{f_{\text{clin}},f_{\text{msr}}}$ values were calculated by multiplying the $k_{Q_{\text{clin}},Q_{\text{msr}}}^{f_{\text{clin}},f_{\text{msr}}}$ factors to the values of field sizes of 5 × 5 and 10 × 10 mm^2^. For each data point, the median value was calculated (Table 2). As shown in Figs. 5 and 6, most EDGE data had larger *OF*_det_ values especially at field sizes of 5 × 5 and 10 × 10 mm^2^. When comparing the mean values of *OF*_det_ and $\Omega_{Q_{\text{clin}},Q_{\text{msr}}}^{f_{\text{clin}},f_{\text{msr}}}$, statistically significant differences were observed (*P* = 0.027, 0.022, 0.014, and <0.01 for 6 MV, 10 MV, 6 MV FFF, and 10 MV FFF beams, respectively). The maximum variations were approximately 15%. After applying the $k_{Q_{\text{clin}},Q_{\text{msr}}}^{f_{\text{clin}},f_{\text{msr}}}$ factors, the variations were significantly reduced. For 6 MV and 10 MV FFF beams, all data were within ±5%. For 10 MV beams, the maximum difference was 5.8%. For 6 MV FFF beams, although one institution showed 7.2% difference, other data were within 4%. For the data measured with the EDGE detector, two institutions submitted the beam data of the TrueBeam with the Millennium 120 MLC (thick lines). The MLC type‐specific difference was not observed.

**Table 2:** Median and 95% confidence interval of $\Omega_{Q_{\text{clin}},Q_{\text{msr}}}^{f_{\text{clin}},f_{\text{msr}}}$ values (output factors corrected with the output correction factors).

FS [mm]	6 MV		10 MV		6 MV FFF		10 MV FFF	
	Median	(95% CI)	Median	(95% CI)	Median	(95% CI)	Median	(95% CI)
5	0.545	(0.531‐0.560)	0.466	(0.456‐0.483)	0.568	(0.559‐0.585)	0.523	(0.513‐0.532)
10	0.708	(0.700‐0.710)	0.670	(0.663‐0.675)	0.718	(0.711‐0.720)	0.710	(0.707‐0.717)
20	0.799	(0.796‐0.801)	0.814	(0.810‐0.817)	0.806	(0.803‐0.808)	0.849	(0.844‐0.853)
30	0.835	(0.832‐0.836)	0.858	(0.856‐0.860)	0.842	(0.839‐0.846)	0.891	(0.888‐0.893)
40	0.868	(0.867‐0.869)	0.891	(0.889‐0.896)	0.879	(0.876‐0.880)	0.920	(0.919‐0.922)
60	0.921	(0.921‐0.922)	0.936	(0.935‐0.936)	0.929	(0.929‐0.930)	0.956	(0.955‐0.956)
80	0.966	(0.965‐0.966)	0.972	(0.972‐0.973)	0.970	(0.969‐0.970)	0.981	(0.981‐0.982)
100	1.000	‐	1.000	‐	1.000	‐	1.000	‐

Abbreviations: CI, confidence interval; FFF, flattening filter‐free; FS, field size defined by multileaf collimator.

## 4 DISCUSSION

In this study, the interinstitutional variability of small‐field dosimetry with the Varian TrueBeam linacs was evaluated. A few studies have reported the inter‐unit variation of TrueBeam data. For example, Glide‐Hurst et al.^23^ compared five TrueBeam machines and reported that the largest coefficients of the variation in *OF*_det_ for field sizes of ≥400 × 400 mm^2^ and 20 × 20 mm^2^ were 0.5% and 1.18%, respectively. Tanaka et al.^25^ evaluated 21 TrueBeam datasets measured for modeling with the Eclipse TPS (Varian Medical Systems) and reported that the relative differences of the *OF*_det_ from the average values were within 1.0% for all field sizes. At a field size of 30 × 30 mm^2^, relative differences were within 0.5%. Imaging and Radiation Oncology Core‐Houston (IROC‐H) previously summarized the measured dosimetric parameters of numerous linacs and reported only very small deviations,^30^ indicating that the variations in dosimetric characteristics of modern machines are very small.

For small‐field dosimetry, however, beam characteristics may vary due to beam‐related causes, such as the focal spot size,^31^, ^32^ detector selection,^10^, ^13^, ^14^ and technical variations of the operators. In this study, the PDD measured with the CC01 ionization chamber had larger values than with other detectors. The under‐responses at shallower depths due to perturbation and volume‐averaging effects resulted in overresponses at deeper depths after renormalization at peak values. Similar results have been reported previously.^18^ For ionization chambers, polarity effects have been reported for measurements of FFF beams.^33^ Although the polarity will not greatly affect the re‐normalized output factors, the effects may be included in the PDD. We also evaluated the OCR for a field size of 10 × 10 mm^2^ and found small variations in the FWHM values. Although the type of the scanning water phantom systems used at each institution was not collected in this study, Akino et al. previously evaluated the PDD and OCR of the same linac using four different scanning water phantoms and reported small variations of <1%.^34^ However, coarse measurement step may affect the OCR parameters including FWHM and penumbra width. For flattened beams, the measurement steps (number of institutions) were 0.5 mm (2), 1 mm (4), and 2 mm (1). For FFF beams, the values were 0.5 mm (1) and 1 mm (2). In this study, the maximum variations in FWHM of flattened beams were larger than those of FFF beams. Although the number of institutions provided OCR data was small, the differences in the measurement step may also have resulted in the uncertainties of the data. The penumbra width of the EDGE and microDiamond detectors seemed smaller than those of other detectors. Tanaka et al.^25^ evaluated the distance‐to‐agreement (DTA) of the penumbra width to the average OCR and reported that the maximum DTA values were within 0.5 mm for a field size of 30 × 30 mm^2^. Their data were collected with an IBA CC13 or PTW 31010 ionization chamber with similar sensitive volumes to compare the collected data with the Representative Beam Data provided by Varian Medical Systems. In this study, the DTA values were calculated from the median OCR profile for each data point (data not shown). Although various detectors were used, the maximum DTA values in the penumbra region defined as 20–80% of the median profile data were 0.50, 0.58, 0.26, and 0.24 mm for 6 MV, 10 MV, 6 MV FFF, and 10 MV FFF beams, respectively, indicating that the variations of the OCR shape were small. At one institution which measured the OCR using a microDiamond, we also measured the crossline and inline OCR profiles at field sizes of 10 × 10 and 20 × 20 mm^2^ using an EDGE detector with both the short and long axes of the detector by rotating the scanning water phantom (data not shown). For all energies and field sizes, the FWHM and penumbrae variations were within 0.3 mm, indicating that the orientation of the EDGE detector does not affect the OCR profile shape. This institution repeated measurements of small‐field dosimetry twice with more than 1‐year interval (Figures [Link], [Link], [Link], [Link]). The difference between two measurements were within 0.3% dose difference (DD) or 0.3 mm DTA for OCR with 10 × 10 mm^2^ field size, within 1.2% DD for PDD of 5 × 5 mm^2^ field size at dose fall‐off region, and within 1.3% difference for *OF*_det_. With appropriate phantom settings, good reproducibility of measurements will be achievable. Although two institutions submitted the beam data of the TrueBeam with the Millennium 120 MLC, only one of them submitted the OCR. To evaluate the impacts of the MLC type on the OCR profiles, further investigations are needed.

In the results of the *OF*_det_, the detector‐dependent effects were clearly demonstrated. The EDGE and unshielded diodes, including Diode E and Diode SRS, had larger responses, compared to the median $\Omega_{Q_{\text{clin}},Q_{\text{msr}}}^{f_{\text{clin}},f_{\text{msr}}}$ value. The maximum variations were approximately 15%. For all beam energies, mean $\Omega_{Q_{\text{clin}},Q_{\text{msr}}}^{f_{\text{clin}},f_{\text{msr}}}$ values were significantly smaller than mean *OF*_det_ values. Most institutions evaluated in this study used diode or diamond detectors whose $k_{Q_{\text{clin}},Q_{\text{msr}}}^{f_{\text{clin}},f_{\text{msr}}}$ factors were negative for field sizes ≤10 mm, although one institution used ionization chamber. When evaluating the *OF*_det_ values collected from multiple institutions, the values will be greatly affected by the detector types used at each institution. After applying the $k_{Q_{\text{clin}},Q_{\text{msr}}}^{f_{\text{clin}},f_{\text{msr}}}$ factors to correct the detector‐dependent effects, the variations were significantly reduced, indicating that the major cause of the interinstitutional variations was detector selection. Although data measured with the same model of detectors showed similar trends, variations remained especially at a field size of 5 × 5 mm^2^. This study was not able to identify the cause of the variations such as machine‐specific, intra‐detector uncertainties, or operator‐dependent effects. If a few auditors conducted onsite measurements for all institutions with their own detectors, inter‐operator and intra‐detector uncertainties will be minimized. Recently, two‐dimensional detector arrays with high spatial resolution using liquid‐filled ionization chambers^35^ or silicon diodes^36^ have become available. These detectors will be helpful because they can acquire *OF*_det_ and OCR concurrently. With sufficient spatial resolution, simplified measurements will minimize inter‐operator uncertainties.

This study included the following limitations. Only 12 sets of corrected beam data were evaluated, which may not be sufficient to generalize the results. Many TrueBeam linacs are clinically used worldwide. In this study, however, data were only collected from institutions that use the TrueBeam with the iPLAN TPS to obtain small‐field dosimetry data acquired with the same measurement protocols. For most of institutions providing beam data in this study, the TPS did not support the Monte Carlo calculation of the FFF beams, resulting in limited OCR for FFF beams. Although the IAEA TRS‐483 recommended to use FWHM for radiation field sizes, we used the setting values of the MLC field sizes instead because of following limitations: (i) not all institutions provided the OCR, (ii) the OCR were measured at 90 cm SSD, whereas the *OF*_det_ values were measured at 100 cm SSD, and (iii) the OCR of 5 × 5 mm^2^ field size were not collected because the data were not required for modelling iPLAN TPS. When increasing the field sizes by 10%, the impacts on the output correction factors of 5 × 5 mm^2^ and 10 × 10 mm^2^ field sizes were less than 0.5% for all detectors. Use of setting field sizes may lead to slight uncertainty, although the impact may be small. In addition, more than half of institutions used EDGE detectors. Because the IAEA TRS‐483 provides the $k_{Q_{\text{clin}},Q_{\text{msr}}}^{f_{\text{clin}},f_{\text{msr}}}$ factors of the EDGE detector only for field sizes ≥8 mm^2^, we calculated the factors for a field size of 5 × 5 mm^2^ by extrapolation. Although the calculated values were close to those in other reports, the IAEA TRS‐483 described that the values of the correction for the small field of interest are limited to a maximum value of 5% in the codes of practice. The extrapolated $k_{Q_{\text{clin}},Q_{\text{msr}}}^{f_{\text{clin}},f_{\text{msr}}}$ factors of the EDGE and CC01 exceeded 5%. We do not recommend the use of the $\Omega_{Q_{\text{clin}},Q_{\text{msr}}}^{f_{\text{clin}},f_{\text{msr}}}$ values shown in Table 2 in clinical practice. However, these results will be helpful to evaluate the validity of the data measured at each institution.

## 5 CONCLUSIONS

We collected the flattened and FFF beam data of the TrueBeam linacs from multiple institutions and evaluated the interinstitutional variability of small‐field dosimetry. Variations were large especially at a field size of 5 × 5 mm^2^. The *OF*_det_ showed detector‐dependent variations and were significantly reduced by applying the $k_{Q_{\text{clin}},Q_{\text{msr}}}^{f_{\text{clin}},f_{\text{msr}}}$ factors, indicating that the interinstitutional variabilities were primarily caused by detector selection. Careful and appropriate detector selection is needed for accurate small‐field dosimetry.

## CONFLICTS OF INTEREST

The authors have no conflict of interest to disclosure.

## Supporting information


**Fig S1.** *OF*_det_: Detector output factors of 6 MV flattened photon beams generated by a TrueBeam with Millennium 120 MLC. $\Omega_{Q_{\text{clin}},Q_{\text{msr}}}^{f_{\text{clin}},f_{\text{msr}}}$: Field output factors calculated as the *OF*_det_ multiplied by output correction factors listed in the IAEA TRS‐483. TPS (treatment planning system) represents the dose calculated by a Varian Eclipse TPS. CC13 and CC04, IBA Dosimetry; EDGE, Sun Nuclear Corp.

**Fig S2.** Beam profiles of 6‐ and 10‐MV flattened photons measured at 100 mm depth with 900 source‐to‐surface distance. MLC field size was 10 × 10 mm^2^.

**Fig S3.** Depth‐dose curves of 6‐ and 10‐MV flattened photons measured with 1000 source‐to‐surface distance. MLC field size was 5 × 5 mm^2^.

**Fig S4.** Detector output factor (*OF*_det_) of 6‐ and 10‐MV flattened photons measured at 100 mm depth with 1000 source‐to‐surface distance.
