# Supplementary figures and images for: Small‐field dosimetry of TrueBeamTM flattened and flattening filter‐free beams: A multi‐institutional analysis
Source: J Appl Clin Med Phys. 2019 Dec 9;21(1):78–87. doi: 10.1002/acm2.12791 (PMC6964782; doi:10.1002/acm2.12791)

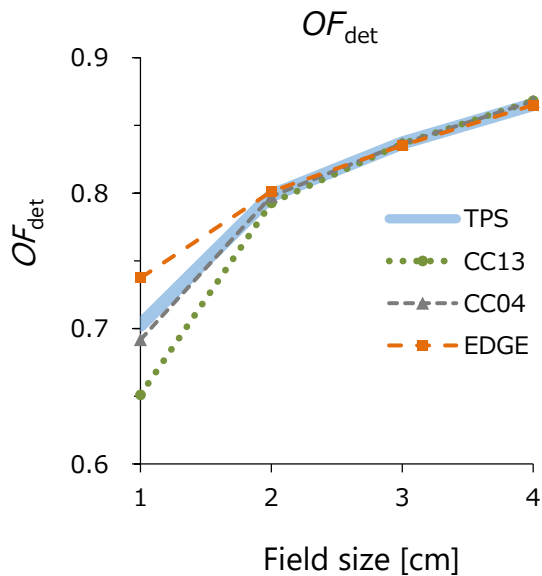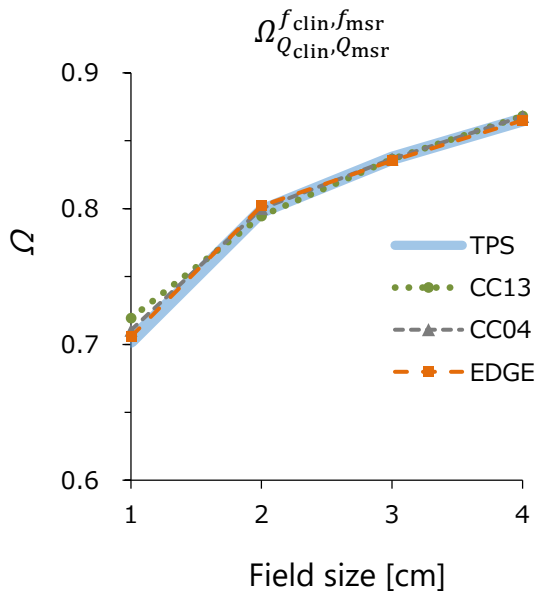

Supplement: Supplementary file 1 — Fig S1. OF det: Detector output factors of 6 MV flattened photon beams generated by a TrueBeam with Millennium 120 MLC. ΩQclin,Qmsrfclin,fmsr: Field output factors calculated as the OF det multiplied by output correction factors listed in the IAEA TRS‐483. TPS (treatment planning system) represents the dose calculated by a Varian Eclipse TPS. CC13 and CC04, IBA Dosimetry; EDGE, Sun Nuclear Corp. [file ACM2-21-78-s001.pdf]

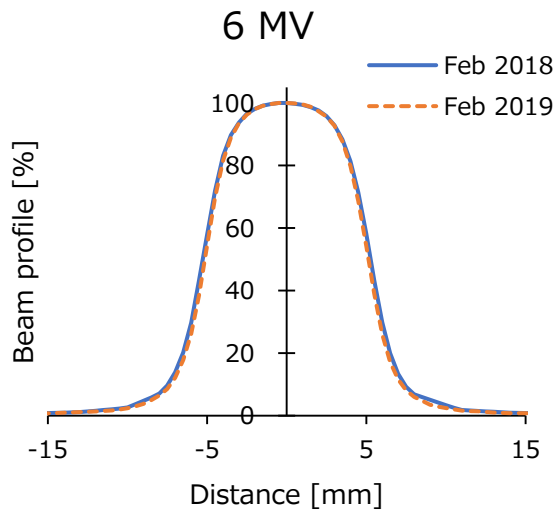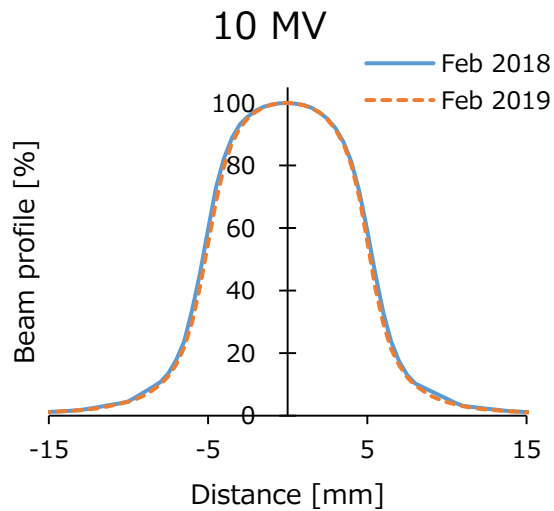

Supplement: Supplementary file 2 — Fig S2. Beam profiles of 6‐ and 10‐MV flattened photons measured at 100 mm depth with 900 source‐to‐surface distance. MLC field size was 10 × 10 mm2. [file ACM2-21-78-s002.pdf]

6 MV

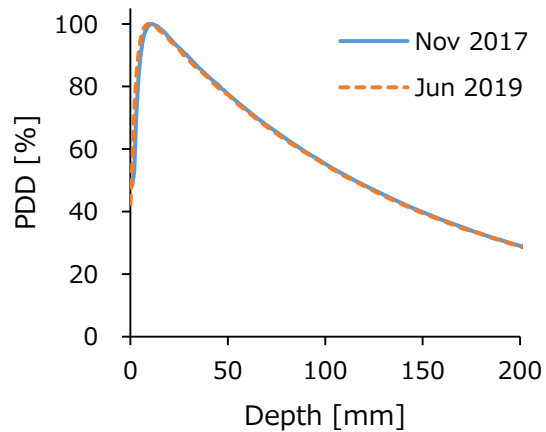

10 MV

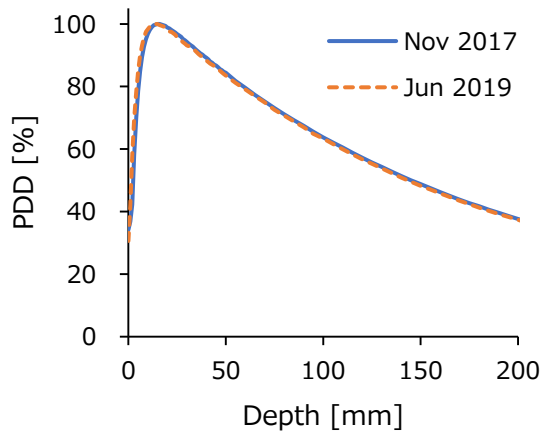

Supplement: Supplementary file 3 — Fig S3. Depth‐dose curves of 6‐ and 10‐MV flattened photons measured with 1000 source‐to‐surface distance. MLC field size was 5 × 5 mm2. [file ACM2-21-78-s003.pdf]

6 MV

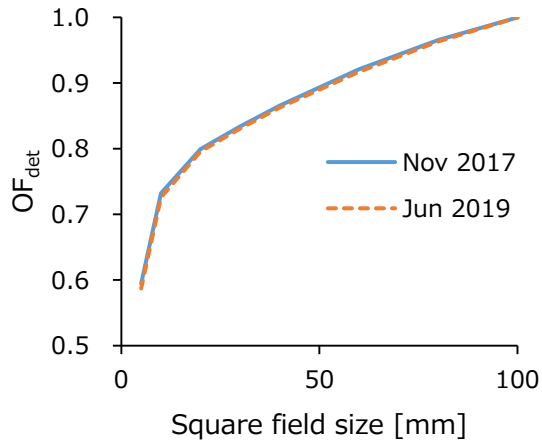

10 MV

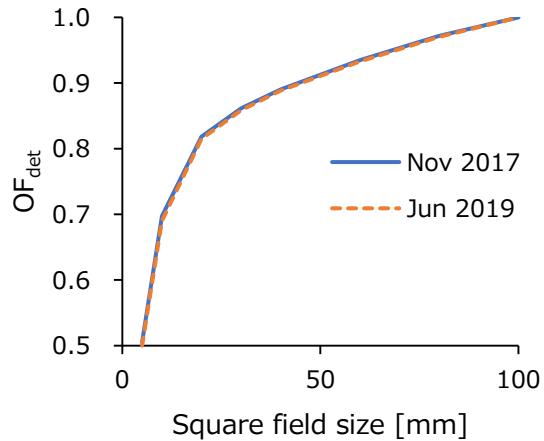

Supplement: Supplementary file 4 — Fig S4. Detector output factor (OF det) of 6‐ and 10‐MV flattened photons measured at 100 mm depth with 1000 source‐to‐surface distance. [file ACM2-21-78-s004.pdf]
